# Australian general practitioners’ perspectives on integrating specialist diabetes care with primary care: qualitative study

**DOI:** 10.1186/s12913-023-10131-4

**Published:** 2023-11-16

**Authors:** Rachael Taylor, Shamasunder Acharya, Martha Parsons, Ushank Ranasinghe, Kerry Fleming, Melissa L. Harris, Deniz Kuzulugil, Julie Byles, Annalise Philcox, Meredith Tavener, John Attia, Johanna Kuehn, Alexis Hure

**Affiliations:** 1 School of Medicine and Public Health, College of Health, Medicine and Wellbeing, University of Newcastle, University Drive, Callaghan, NSW 2308 Australia; 2 Hunter Medical Research Institute , New Lambton Heights, NSW 2305 Australia; 3 Hunter New England Health District, John Hunter Hospital, NSW, Lookout Road, New Lambton Heights, 2305 Australia

**Keywords:** Diabetes, Qualitative analysis, Primary care, General practitioners, Service integration

## Abstract

**Background:**

Improving the coordination and integration of health services is recognised nationally and internationally as a key strategy for improving the quality of diabetes care. The Australian Diabetes Alliance Program (DAP) is an integrated care model implemented in the Hunter New England Local Health District (HNELHD), New South Wales (NSW), in which endocrinologists and diabetes educators collaborate with primary care teams via case-conferencing, practice performance review, and education sessions. The objective of this study was to report on general practitioners’ (GPs) perspectives on DAP and whether the program impacts on their skills, knowledge, and approach in delivering care to adult patients with type 2 diabetes.

**Methods:**

Four primary care practices with high rates of monitoring haemoglobin A1c (HbA1c) levels (> 90% of patients annually) and five practices with low rates of monitoring HbA1c levels (< 80% of patients annually) from HNELHD, NSW provided the sampling frame. A total of nine GPs were interviewed. The transcripts from the interviews were reviewed and analysed to identify emergent patterns and themes.

**Results:**

Overall, GPs were supportive of DAP. They considered that DAP resulted in significant changes in their knowledge, skills, and approach and improved the quality of diabetes care. Taking a more holistic approach to care, including assessing patients with diabetes for co-morbidities and risk factors that may impact on their future health was also noted. DAP was noted to increase the confidence levels of GPs, which enabled active involvement in the provision of diabetes care rather than referring patients for tertiary specialist care. However, some indicated the program could be time consuming and greater flexibility was needed.

**Conclusions:**

GPs reported DAP to benefit their knowledge, skills and approach for managing diabetes. Future research will need to investigate how to improve the intensity and flexibility of the program based on the workload of GPs to ensure long-term acceptability of the program.

**Supplementary Information:**

The online version contains supplementary material available at 10.1186/s12913-023-10131-4.

## Introduction

The rising prevalence of type 2 diabetes (T2D) is a significant concern to healthcare systems worldwide. In 2017, it was estimated that 462 million adults (6.28% of the world’s population) were affected by T2D [1]. However, 46% (or 174.8 million) of all diabetes cases are estimated to be undiagnosed, therefore the prevalence of the condition is likely to be underestimated [2]. In Australia, one million adults (5.3% of those aged 18 years and older) were diagnosed with T2D in 2017-18 [3]. Poorly controlled diabetes is associated with an increased risk of hospitalisation and health complications including macrovascular disease, lower limb amputations, blindness, kidney failure, and premature death [4, 5]. Diabetes represents 11% of all hospitalisations in Australia and 64% of these cases are due to T2D [6]. Diabetes is the third largest chronic health condition for hospitalisation expenditures related to cases classified as potentially preventable [7]. The average annual healthcare cost per person with diagnosed diabetes is $4,390; and the cost for those with micro- and macrovascular complications is 2.2 times higher compared to those without complications [6].

In the Australian primary healthcare system, at least one third of patients do not receive care consistent with best practice guidelines [7]. Significant barriers have been identified in the delivery of quality diabetes care including limited resources and organisational capacity [8, 9], time and workload pressure [8, 10], and a lack of knowledge and skills related to diabetes management [11, 12]. In response to the rising prevalence of diabetes and barriers associated with providing quality care, the Australian National Diabetes Strategy (2016–2020) was established [13]. The strategy identified several key principles for policies and programmes that address current gaps, which include better coordination and integration of services, patient-centered care and management, and improved measurement of health behaviours and outcomes [13]. Based on these principles from the national strategy, the Diabetes Alliance Program (DAP) was developed and implemented in the Hunter New England Local Health District (HNELHD) in 2015 as a new model of integrated care. The DAP is a collaboration between hospital specialists and primary care teams to provide capacity-enhancing case-conferencing with patients, as well as education, and performance monitoring and feedback, as previously described [14]. Case conferencing involves hospital specialist teams (endocrinologist and diabetes educator) visiting primary care practices to participate in a series of 40-minute consultations involving the patient, and their general practitioner (GP), and the practice nurse. GPs are recognised as the main diabetes care provider over the longer term and the practitioner who knows and understands their patient’s needs and circumstances. During the clinics, case conferencing is led by the Staff Specialists for the purpose of upskilling; however, this is done in a collaborative and consultative way to maximise the benefits for the patient seeing a multidisciplinary team and to build trust across healthcare providers. During the consultation, diabetes complications and comorbidities are reviewed and the treatment plan is discussed. Prior to case conferencing, approximately 30–60 min of preparatory work is completed by practice nurses and Primary Health Network practice support officers, which include organising podiatry and eye review, up-to-date pathology, and completing a diabetes clinical information sheet. Six months after the initial case conference, patients are reviewed by their GP and the hospital specialist team. The administrative staff of the primary care practice are responsible for arranging and booking patients for case conferences. This model of care is supported through a Service Level Agreement between the HNELHD and the Primary Health Network Hunter New England and Central Coast. Medicare billing items were applied for case-conferencing.

Qualitative findings indicate that the patient experience with DAP are positive and the program is beneficial for improving self-management skills for diabetes [15]. Furthermore, recent quantitative findings indicate that DAP is associated with spillover improvements in clinical outcomes for all patients with T2D [16, 17]. Internationally, integrated healthcare delivery for the management of diabetes has been associated with a reduction in referrals for specialist care [18], improvement in clinical outcomes [19] as well as mental health [20]. A systematic review of 16 studies including nine randomised controlled trials found that integrated models of care in the United States were effective in managing diabetes in the primary care setting [21]. To date only three Australian studies have explored the experiences and views of GPs’ located in Sydney, Melbourne and Brisbane towards integrated models of care for the management of diabetes in primary care [22–24]. To our knowledge this is the first Australian study to analyse the views of GPs based in the HNELHD. This data will be important for understanding the acceptability of this model of care by GPs and informing future refinements of the program. Therefore, the current study aims to report GPs perspectives on DAP, in particular perceptions of impact on knowledge, skills and approach in managing care for adult patients with T2D.

## Methods

### Study design

To explore GPs’ experiences and perspectives of the DAP, we conducted semi-structured interviews and analysed themes [25, 26]. Practice nurses were also interviewed, with findings being collated separately. The reporting of this qualitative study was in accordance with the 32-item Consolidated Criteria for Reporting Qualitative Research checklist (COREQ), designed for interviews and focus groups [27].

### General practitioner sampling frame

GPs interviewed for this study were selected based on the percent of patients within their practice who had a diagnosis of T2D and the rate at which they completed an annual HbA1c test. Four practices, with four GPs, were classified as ‘high performing’, and five practices, with five GPs, were classified as ‘low performing’. Practices were classified as ‘low performing’ if less than 80% of their patients diagnosed with T2D had their HbA1c levels measured in the last 12 months prior to being involved in the DAP, and ‘high performing’ if more than 90% of their patients with T2D had their HbA1c levels measured in the last 12 months. Sample size was determined by thematic saturation in which concurrent analysis revealed that no new themes and no new insights had emerged from successive interviews [28]. When all researchers (MP, US, MLH, DK) came to consensus that saturation had been reached, recruitment and interviewing ceased.

### Setting, participants and recruitment

Interviews were conducted with GPs from primary care practices in the HNELHD. GPs that had participated in DAP were approached by the Program Manager and provided with a formal letter of invitation and participant information statement for the study. Ethical approval was granted for all aspects of the project by the Hunter New England Human Research Ethics Committee (15/04/15/5.02) and all GPs gave written informed consent prior to the commencement of the interview.

### Data collection

The semi-structured interviews with the GPs were conducted by one of two Endocrinology advanced Trainees (DK or UR). The interviewers disclosed their position and prior involvement in DAP prior to obtaining consent from GPs. Prior to data collection, the interviewers were trained in conducting interviews by conducting a practice session with the program manager (MP). Face-to-face interviews were conducted with the GPs at their practice site and were digitally recorded; only the interviewer and GP were present during the interview. Interviews were guided by the interview schedule which contained a topic list of open-ended and probing questions (Additional File 1). The interview schedule was developed through discussion with the DAP team. One pilot interview was conducted to test the length, flow, and clarity of the questions and to refine any questions that were unclear based on the feedback from the session. Data from the pilot interview were not included in the current analysis. At the start of the interview, GPs were reminded that they could stop or pause the interview, or withdraw consent at any time for any reason without jeopardising their participation in DAP. During the interview, GPs were also able to direct the conversation within the topics and explore related issues in greater depth. Interviews were conducted between August 2018 and November 2019 and ranged from 30 to 45 min in duration. No interviews were repeated. Interviews were audio recorded and transcribed. The interview transcript was not returned to GPs for comment or correction.

### Data analysis

Transcripts were de-identified and reviewed for accuracy (UR, KF). Coding and analyses were completed (UR, KF) using the Nvivo qualitative analysis program (v.12) (QSR International, Melbourne; Australia). Data analysis was based on pragmatic themes to assess patterns and meaning in the data. To summarise, transcripts were closely read by one researcher to understand the overall content of the data. After completing all interviews, initial data coding was generated by one researcher that read all transcripts line by line, with sections of text identified for emergent patterns which were identified in Nvivo as free-standing nodes. These initial nodes reflected the responses of the GPs in their own words and were defined using descriptive labels for the codebook. All transcripts were then iteratively reviewed and analysed to ensure the accuracy of identified themes. The themes were further refined by confirming if the generated codes were associated with coherent patterns within and across the data while also searching for disconfirming evidence [29]. All GPs were given a pseudonym in the reporting of findings from the interviews to maintain anonymity.

## Findings

### Characteristics

Of the nine GPs that were invited to participate, no GPs declined or were not available to be interviewed.

The results presented were based on the following themes:

1. Overall impression of case-conferencing.
2. Knowledge and skills of GPs.
3. Approach to diabetes care.
4. Room for improvement.

### Theme 1) overall impression of case-conferencing

Overall, GPs described DAP as *“quite helpful”* and a *“very educational”* experience. One GP felt that observing case-conference conversations was a major asset within the program, stating that *“To be… an observer… during the [case-conference] conversation [and] watch the management be discussed by the team is good” (GP3).* Beyond improving knowledge of GPs, the program was also viewed as valuable for improving care for patients. One GP felt that the program supported positive relationships with patients and commented that *“we have a very good rapport” (GP8).* GPs were supportive of the structure and delivery of the program which they felt would improve the accessibility of multidisciplinary care for patients. For instance, GP5 stated that, *“it was convenient for the patients… [to access] services like a dietitian and endocrine specialists at the same place.”* This was echoed by GP7 who said that *“the accessibility… is fantastic… [patients] can see a consultant in their own surgery where they feel comfortable with their own doctor, so they feel like they [are] getting special care.”*

### Theme 2) knowledge and skills of GPs

#### Increased confidence with medication prescribing and monitoring

GPs reported that they had not previously started patients on insulin medication and would usually refer patients to the hospital specialist team to provide that care. However, since participating in the program these GPs felt they were significantly *‘upskilled’* and were confident to prescribe insulin therapy. DAP assisted GPs to change and evolve by using the latest insulin analogues and this was described as a key asset of the program. One GP (GP7) described that the education about new medications received during the program was essential for understanding *“where they fit, when to change, what [are] the criteria…”* GPs were generally more confident in making significant changes to patient medications and were most likely to escalate treatment. For instance, GP7 felt that *“I probably was more… reluctant to change all medications at once- I [would usually] just [change] one over three months… Whereas now if I want to change, I’ll change them all at once.”* GPs also demonstrated increased skills in prescribing medications with greater capacity to use different combinations of medications and provide individualised treatment plans. As stated by GP2 *“I’m not afraid to change between different medications.”*

#### Knowledge and skills transfer

GPs acknowledged that the education received during the program impacted the provision of care provided to patients with diabetes who were not involved in DAP. GP3 described their intention to *“implement some of the newer drugs”* with other patients with diabetes. Assessing lifestyle factors (e.g. diet) and providing education about the importance of lifestyle factors for diabetes management was also implemented with other patients. For instance, GP4 reported that, *“there’s a few [GPs] that… have taken a step back to look at their [patient’s] diet.”*

As a result of participating in DAP, GPs had a heightened awareness of the importance to screen for pre-diabetes in all patients. This was indicated by GP7: *“We try to… [find] out if the patient is pre-diabetic [so they can be referred] to the dietitian, exercise physiologist…”* Instances were described of increased diabetes assessment and monitoring with other patients. For instance, GP8 commented that: *“Every time now [I measure] body weight [for] all diabetic patients. [as well as] …blood pressure.”*

### Theme 3) approach

#### Holistic approach to care

Since participating in DAP, GPs had noticed a change of practice towards dealing with *“the whole package, not just their diabetes” (GP3)* and therefore providing a more holistic approach to care. GP1 recognised that the program had increased their awareness to screen for other co-morbidities associated with diabetes:

… there were 4 or 5… of my patients that we suggested to have sleep apnoea tests… so it’s… a global view of everything that… might be at risk, not just the diabetes.

Rather than solely focusing on adjusting medications to manage diabetes, GP3 stated: “*I’m… [now] looking at their [physical] activity levels and their diet”* before making any decisions about the management plan.

#### Changes to referral pathways

Due to involvement in the program GPs felt *“more comfortable in managing diabetes”* within their practice and were not referring patients for tertiary care as often. GP9 also commented: *“I am not even referring as many private patients to… endocrinologists.”* Although there were also special circumstances in which a referral to a specialist was required as indicated by GP9: “*Most of the… [patients] that I’m referring now are ones [patients] that I have to refer because of… commercial driver’s licence requirements or train driver requirements and things like that.”* It must be noted that although the majority of GPs described making fewer referrals to specialist care, one GP (GP6) felt that the program had not significantly changed the number of referrals that they made for patients.

GPs shared that the program also helped them to establish an investigative process for assessing patients with uncontrolled diabetes to identify and address the problem before referring the patient. This was demonstrated by GP6:

I probably wouldn’t have tried to identify what was going on first… now if we [GPs] have anybody [patients] who is a bit out of control [for managing their diabetes] I do a routine blood test to make sure they’re not septic. I’ve got my little routine [and an assessment checklist such as] are they testing their sugars properly, are they… giving their insulin properly… but if we’re not getting places [in addressing the problem] then I’d refer on [the patient to the endocrinologist].

### Theme 4) room for improvement

All GPs agreed that aspects of case-conferencing could be improved. The preparation involved for case-conferences was considered intense, especially when administrative support was limited within the primary care practice. This was reported by GP5: *“it consumed a lot of time for the practice because we don’t have a practice manager… so we had to do a lot of things by our self.”* GP5 felt that *“decreasing or minimising”* the paperwork would improve their experience with case-conferencing. One GP (GP3) also felt that conducting case-conferencing needed to be more flexible based on their clinical workload. GP3 indicated that:

[when] we’ve got a morning of alliance [DAP] patients [that will] pretty much… write off a morning of general practice or at least half a morning… [It would be better to have] a little bit more freedom to come and go during the process if there are other things you have to deal with in the practice.

One GP (GP4) also expressed that case-conferencing needed to be adaptable based on the needs of the patient. This was suggested by GP4 *“some patients didn’t need as much time as we allocated so we could… be seeing more [patients] in the clinic.”* Case-conferencing was regarded as an informal process and could be improved by applying a more structured approach. This was expressed by GP6:

“I thought maybe more structure can be given to the [case-conference] meetings by formulating the problem areas [of the patient] before the meeting… [and] the specialist could be the chairman…. I think if you have a meeting somebody should be making… notes and everybody of the team should get… a summary of the notes.”

Two GPs (GP2 and GP7) also felt that more time was needed between case-conferencing, especially if significant changes were made to the patient medication regime. The use of alternative communication methods such as video case-conferencing rather than in-person clinic visits, as well as email contact between case-conferences, was also suggested by two GPs (GP7 and GP8).

## Discussion

This study provides supporting evidence that an integrated diabetes care model is beneficial for increasing the skills and confidence of GPs in delivering best practice and evidence-based care for diabetes management. Evidence from studies conducted in Australia [30, 31] and internationally [32, 33] have shown that integrated healthcare delivery in primary care is cost-effective compared to usual models of care.

Some GPs reported that the additional time taken for the preparation of case-conferences can be burdensome; however, it seemed that much of that time was needed for updating pathology, eye, and podiatry reviews which is a routine part of diabetes care. Similarly, other qualitative studies have identified that the additional administrative work, staffing limits and time pressures were significant barriers for the implementation of integrated models of care [23, 24, 34]. If practices implement systems and processed that support adherence to The Royal Australian College of General Practitioners (RACGP) guidelines and regular annual care cycles, less time would be required to prepare for case-conferences.

GPs expressed higher levels of confidence and skills for providing diabetes care as a result of the education received during DAP. Most of the interviewed GPs reported making less referrals to specialist care as their capacity to manage a patient’s diabetes care increased. This is in agreement with the findings of Walsh et al. [18] that after participating in a one year community diabetes initiative there was a 31% reduction (p < 0.01) in the total number of referrals to specialist diabetes clinics and a 57% reduction (p < 0.01) in referral outside the referral guidelines by GPs in the United Kingdom. This is a significant improvement since diabetes is the fourth most frequently referred health problem; it is estimated that Australian GPs make 4.5 referrals to specialist care per 100 cases of diabetes encountered [35]. Increasing the management of diabetes in the primary care setting will impact the provision of health services by enabling high risk patients to be prioritised for specialist care.

A lack of confidence, knowledge, and skills have been identified as key barriers for initiating injectable therapy including insulin in primary care [11, 36]. The DAP was able to address these key barriers and consequently GPs felt significantly more confident in their skill level in commencing insulin medications and making adjustments to medication regimes. This is significant for diabetes care since a one year delay in treatment escalation in patients with uncontrolled T2D is associated with an increased risk of cardiovascular complications [37]. Therefore, the skills that the GPs gained from the program are likely to prevent downstream adverse consequences as a result of timely therapeutic escalation.

This study provided valuable and rich data for understanding the acceptability of the DAP in primary care practice and whether participation in the program was associated with improvements in knowledge, skills, and approach of GPs. The outcomes achieved by GPs in the program are largely influenced by the relationship, trust and communication that is established between the hospital specialist team and GP, which is supported by the Relational Coordination Theory [38]. This study sampled GPs from high and low performing primary care practices to ensure diversity in the sample and experiences of the practitioners included in this study. Despite these strengths, the study findings should be interpreted in light of the following limitations. Sampling bias cannot be excluded as GPs volunteered and consented to participate therefore, the sample may be representative of those that were more engaged and are possibly more favourable towards the program.The sample size was limited to nine GPs and therefore the findings presented may not reflect the broad and vast perspectives of GPs enrolled in the program. The translatability of this model of care to other locations nationally and internationally is unknown. It is likely that the model would require adaptation for rural and remote settings due to travel distances between primary and tertiary care. The intensity and administrative burden should be considered in the implementation of the model in other similar settings. Furthermore, this model of healthcare of delivery may be relevant to other chronic health conditions and should be investigated by future studies.

An overall goal of DAP is to improve the knowledge, skills and confidence of Primary Care Clinicians in the delivery of evidence-based care for adults with diabetes. Findings from this study provide supporting evidence that the program is accomplishing this goal and GPs have favourable views towards the program. Time and administrative burden were acknowledged as barriers for delivery of the program. However, DAP has been shown to be highly valued by patients [15] and the program has contributed to significant health benefits for patients not directly involved in the program [16, 17]. Therefore, the demonstrated benefits of DAP outweigh the identified barriers for delivery the program. DAP has been implemented as been business as usual since 2017 for HNELHD and there are plans for scale up and implementation in other local health districts in New South Wales and beyond.

This integrated model of care was positively received by GPs for improving diabetes care in primary care practice. Participation in DAP was associated with GP self-reported improvements in knowledge and skills, a more holistic approach to care, changes in referral pathways, and increased confidence in prescribing medications. Improvements in diabetes care provided by GPs was also transferred to patients with T2D that were not directly involved in the program. Feedback from the GPs indicated that the program could be further improved by reducing the administrative workload and time involved in conducting case-conferencing.

### Electronic supplementary material

Below is the link to the electronic supplementary material.

Supplementary Material 1

## Data Availability

The author confirms that all data generated or analysed during this study are included in this published article.

## Abbreviations

COREQ: Consolidated Criteria for Reporting Qualitative Research checklist
DAP: Diabetes Alliance Program
HNELHD: Hunter New England Local Health District
GPs: General practitioners
RACGP: Royal Australian College of General Practitioners
T2D: Type 2 diabetes (T2D)
