# Supplementary material for: Australian general practitioners’ perspectives on integrating specialist diabetes care with primary care: qualitative study
Source: BMC Health Serv Res. 2023 Nov 16;23:1264. doi: 10.1186/s12913-023-10131-4 (PMC10652609; doi:10.1186/s12913-023-10131-4)
Supplement: Supplementary file 1 — Supplementary Material 1 [file 12913_2023_10131_MOESM1_ESM.docx]

# **Additional File 1**

# **Qualitative Interview Schedule**

## **General Practitioners**

Insights gained from the audit, training and case-conferencing.

**Today I’d like to talk to you about the case conferencing process. I would like to get a sense of your thoughts and feelings about what your practice was like before and after the program was instigated. Firstly, could you start by describing your experience of being involved in the Alliance program?**

- Do you feel you have learnt anything about diabetes care through your involvement with the Alliance Program? Could you tell me more about that? Do you feel your prescribing practices have changed?
- Has your involvement in the Alliance Program changed the type of patient or number of patients you refer to a diabetes specialist? Could you tell me more about that?
- Has your involvement in the Alliance Program changed how you manage the care of your other patients with diabetes that weren’t directly involved in the program? Could you expand on that?

Whether and how the program has changed the way the GP cares for people with diabetes.

- How has your attitude toward the management of patients with diabetes changed after your involvement in the program?

Whether and how the case conferencing has been helpful, and ways in which the process could be improved.

**As part of this evaluation process, we would like to understand what parts of the program worked really well for you and what parts you think could be improved. Could you tell me about what aspects of the program you liked the most?**

**Could you tell me about what aspects of the program could be improved?**

- Do you think a patient’s involvement in the program changes their approach to and understanding of their diabetes? If so, how?
- Have you found an individual patient’s involvement in the Alliance Program affects their overall engagement and understanding of their diabetes care?
- What do you think is the main barrier for optimizing diabetes control in primary care settings?
- How did you find the amount of preparation required for case conferencing?
- How did you find the medicine insight program?
- Did you find the Alliance data feedback help? If so, how?
- Did you find the Alliance Master Class education sessions help? If so, how?
- Did you find the real life case conferencing help? If so, how?
- Can you suggest any ways the case conferencing could be improved?

**Before we finish up the interview, I’d just like to ask, is the Alliance program something you would recommend to other GPs? Is there anything you would like to add that we haven’t already discussed?**

**Thank you for your time today.**
